# Inorganic Macro- and Micronutrients in “Superberries” Black Chokeberries (*Aronia melanocarpa*) and Related Teas

**DOI:** 10.3390/ijerph14050539

**Published:** 2017-05-18

**Authors:** Iva Juranović Cindrić, Michaela Zeiner, Darija Mihajlov-Konanov, Gerhard Stingeder

**Affiliations:** 1 Division of Analytical Chemistry, Department of Chemistry, Faculty of Science, University of Zagreb, Horvatovac 102a, 10000 Zagreb, Croatia; ijuranovic@chem.pmf.hr (I.J.C.); darija1512@gmail.com (D.M.-K.); 2 Department of Chemistry, University of Natural Resources and Life Sciences, Muthgasse 18, Vienna 1190, Austria; gerhard.stingeder@boku.ac.at

**Keywords:** *Aronia melanocarpa*, berries, infusions, mineral content, extraction yield

## Abstract

Black chokeberries (*Aronia melanocarpa*) are considered to be functional food containing high amounts of anthocyanins, phenols, antioxidants, vitamins and minerals. Whereas organic compounds are well studied, there is little research on the mineral composition of the chokeberries. Thus, the presented study is focused on the determination of Al, As, Ba, Ca, Cd, Co, Cr, Cu, Fe, K, Li, Mg, Mn, Mo, Na, Ni, Pb, Se, Sr and Zn in black chokeberry fruits and infusions to study the metals’ extractability. The nutrients Ca, K and Mg are present in the fruits (dried matter) at g/kg level, whereas the other elements are present from µg/kg up to mg/kg level. The extraction yields of the metals from the infusion range from 4 (Al, Mn) up to 44% (Na). The toxic elements present do not pose any health risk when berries or infusions are consumed. Concluding, Aronia berries, as well as infusions derived from them, are a good dietary source of essential metals in addition to the organic compounds also contained.

## 1. Introduction

*Aronia melanocarpa* (black chokeberry), belonging to Rosaceae family, originally coming from North America is now widely used also in Europe as a “superberry” due to its high content of nutrients being especially beneficial for health and well-being. Not only do scientific papers describe the beneficial properties of this berry [1], but this fruit is also popularized in non-scientific journals [2]. The Aronia plant can reach a height of 2 to 3 m. The fruits, dark because of a high amount of anthocyanins, have a diameter of 6–13 mm and weigh between 0.5 and 2 g. The ripe berries are usually harvested in August and September. Aronia is nowadays commonly used in different European countries as berries themselves or processed as syrup, juice, jellies and tea [3]. The fruits are rich in polyphenols and have thus been intensively investigated for its medicinal potentials. Besides the antioxidative activity [4], Aronia contains components such as isoquercetin, kaempferol, ferulic acid, caffeic acid, ellagic acid, myricetin and hydroxybenzoic acids, which all have been found to exhibit anti-influenza activities [5]. Cytotoxic effects against cervix carcinoma, melanoma, colon, and chronic myelogenous leukemia cell lines have been correlated with the phenolic constituents [6] while anthocyanins extracted from chokeberry juices provide positive effects against colon cancer [7,8]. Due to these biological activities chokeberries are considered as a functional food. Compared with other berries, Aronia berries contain a lot of cyanidin glycosides, only beaten by elderberry [9] and bilberry [10].

While a large number of scientific publications focus on the organic compounds in the berries responsible for different medicinal activities, only few reports on the inorganic pattern of these berries, either on toxic or on essential elements, were found [3,11]. Berries in general are a rich source of macro- and microelements [12]. Already in the 1940s, Chandler stated that berries have unsurpassed ability in accumulating essential elements among fruiting plants [13]. Thus, not only is the metal content of the berries themselves is of interest, but also their extraction yield too, since berries are widely consumed as infusions or juices. 

Furthermore, it has been proven that certain elements enhanced the production of the flavonoids leading to the beneficial effects of berries [14,15]. The dietary intake of chokeberries can also be promoted due to the metal ion binding capacity of its fibers. Whilst Mg is desorbed under digestive conditions, harmful elements, such as Cd, Pb, Cu, Zn are absorbed [16,17].

Thus, the main objective of the present paper is the determination of harmful and beneficial elements in berries and infusions prepared from them in order to get a complete picture of the berries’ composition.

## 2. Materials and Methods

### 2.1. Chemicals and Glassware

Nitric acid (suprapure) and inductively coupled plasma (ICP) Multi-element Standard IV were purchased from Merck (Darmstadt, Germany). The standard reference material of strawberry leaves (LGC7162) used was obtained from LGC Standards (Middlesex, London, UK).

All plastic (PE) and glassware was cleaned with diluted nitric acid prior to use. Ultrapure water was produced by an in-house water preparation system.

### 2.2. Samples

Aronia berries (*n* = 11) were purchased from local markets for organically grown food in Croatia (2011), dried for 24 h at 105 °C, homogenized in a metal free mortar and stored in a dry and dark room at ambient temperature prior to digestion.

### 2.3. Sample Preparation

All plant samples were digested using a closed microwave assisted system, as it is state of the art for such samples [18].

For the sample preparation, a MWS-2 Microwave System Speedwave from *Berghof* Laborprodukte GmbH (Eningen, Germany) was used. Each sample (berries and CRM) was digested in duplicate, whereby about 0.3–0.5 g (weighed to the nearest 0.1 mg) was put into a Teflon reaction vessel and 5 mL HNO_3_ (50:50 *v/v*) were added. The digestion procedure was carried out according to the following program consisting of three steps of 15 min each: 110 °C–170 °C–140 °C. Blank solutions were prepared in the same way.

The clear solutions were obtained and then brought to final 10.0 mL with ultrapure water.

The infusions were prepared by addition of 98 mL boiling ultrapure water to 2.0–2.4 g (weighed to the nearest 0.1 mg) of dried berries. Different decoction times were applied, namely 1, 5, 10, 20 and 30 min. The final infusions were filtered using Whatman No 541 filter paper (Merck; Darmstadt, Germany) in order to have clear solutions for the measurements by inductively coupled plasma—atomic emission spectrometry (ICP-AES).

### 2.4. ICP-AES Measurements

The elemental concentrations in the digests and the infusions were measured in triplicate by a Prodigy High Dispersive ICP-AES spectrometer from Teledyne Leeman (Hudson, NH, USA) working in a simultaneous mode. The optimal instrumental conditions are listed in Table 1 and the emission lines used were: Al 308.215 nm, Ba 455.403 nm, Ca 396.847 nm, Cd 214.441 nm, Co 228.615 nm, Cr 206.149 nm, Cu 224.700 nm, Fe 238.204 nm, K 766.491 nm, Mg 280.271 nm, Mn 257.610 nm, Na 589.592 nm, Ni 231.604 nm, Pb 220.353 nm, Sr 407.771 nm, and Zn 213.856 nm.

The quantification was done by external calibration using multi-element standard solutions in the concentration range from 0.05 mg/L to the expected maximum concentration ranging from 5 to 20 mg/L of the elements of interest. The standard solutions were prepared by dilution of a multi-element standard stock solution (1000 mg/L) with 1% *w/w* HNO_3_. Blank solutions were prepared in the same medium.

### 2.5. Inductively Coupled Plasma—Mass Spectrometry (ICP-MS) Measurements

Additionally, the elements were quantified with high-resolution inductively coupled plasma—sector field mass spectrometry (ICP-SFMS) (Element 2 ICP-SFMS from Thermo Fisher; Bremen, Germany). The instrument was equipped with a self-aspirating PFA microflow nebulizer (ESI: Elemental Scientific Inc., Omaha, NE, USA; flow of 100 mL/min), a Peltier cooled (PC^3^) cyclonic quartz chamber (ESI: Elemental Scientific Inc., Omaha, NE, USA; operated at 4 °C), a quartz injector pipe and torch, aluminum sampler and skimmer cone (all from Thermo Fisher). The instrumental conditions applied were: radio frrequency (RF) power-1300 W and plasma gas flow-16 L/min, sample gas-1.06 L/min and auxiliary gas flows-0.86 L/min.

The isotopes were analyzed at different resolutions, low resolution (^7^Li^+^, ^82^Se^+^, ^88^Sr^+^, ^111^Cd^+^, ^208^Pb^+^), medium resolution (^52^Cr^+^, ^55^Mn^+^, ^56^Fe^+^, ^59^Co^+^, ^60^Ni^+^, ^65^Cu^+^, ^66^Zn^+^, ^98^Mo^+^) and high resolution (^75^As^+^), with the nominal mass resolutions of 350, 4500 and 10,000, respectively. At all resolution levels Indium (1.1 μg/L) was used as internal standard, measured as ^115^In^+^.

### 2.6. Optimisation and Characterisation of the Analytical Method

The trueness of the method for digests was determined by analyzing strawberry leaves reference material. For the infusions, spiking experiments were performed to determine the recovery: aqueous multi-element standard solutions were added to a set of infusions in triplicate at three concentration levels (1.0, 3.0 and 5.0 mg/L) according to the expected concentration range.

The overall repeatability of the instruments was determined by analyzing three samples after calibration on two different days. The precision as relative standard deviation (RSD) was evaluated by measuring certain samples five times.

The limits of detection (LOD) were calculated according to Boumans [19] using 3σ.

## 3. Results and Discussion

### 3.1. Analytical Methods

The results from the blank solutions (digest solution, infusion blank) are subtracted from the sample values, so all the data given in the following are blank corrected.

The trueness, describing how close the test result is to the true result, is expressed by recoveries determined by analyzing the standard reference material of strawberry leaves (LGC7162) and ranged from 85 up to 109% for both analytical methods (Table 2). The recoveries were calculated according to the following formula:

$$recovery_{x}=\frac{{\mathrm{content}}_{x,\;\;found}\;\left[\mathrm{mg}/\mathrm{kg}\right]}{{\mathrm{content}}_{x,\;\;certified}\;\left[\mathrm{mg}/\mathrm{kg}\right]}\;\times \;100$$

The spiking experiments for the infusions resulted in recovery rates from 92 to 106% for ICP-AES (Table 3), whereby the recoveries were calculated according to the following formula:

$$recovery_{x}=\frac{{\mathrm{concentration}}_{x,\;\;spiked\;solution}\;\left[µg/L\right]-{\mathrm{concentration}}_{x,\;\;unspiked\;solution}\;\left[µg/L\right]}{{\mathrm{concentration}}_{x,\;\;added}\;\left[µg/L\right]}\;\times \;100$$

High particulate matter in the infusions even after an additional centrifugation step caused plugging of the ICP-MS tubes, so the infusions could not be measured by ICP-MS. Thus, there are no results for As, Li, Mo and Se.

The LODs in the digested berries were below 1 mg/kg for ICP-AES and up to 20 µg/kg for ICP-MS for the elements studied (see Table 4). The LODs determined for the metals in infusions are presented in Table 5.

Regarding the calibration curves, the coefficients of determination (R^2^) were higher than 0.9990.

The precision determined by repeated measurements led to RSD values in the range from 0.02 up to 1.8%, while the day-to-day repeatability for all metals was below <1.9%.

All these obtained analytical parameters are in the range for the determination of micro- and macro-elements in biological samples.

### 3.2. Elemental Analysis of Berries

Elemental composition of Aronia berries is listed in Table 4 as mean values along with the respective standard deviation of *n* = 11. Arsenic, Ba and Se could not be detected in any samples. Furthermore, literature data [11,20,21] for minerals in Aronia berries are presented in Table 4. All three publications are focused only on few elements and in all cases the contents are determined in fresh fruits. Due to differences in water content in berries, such data can be used only for rough comparison. It can be clearly seen that the obtained results here are higher, since they are calculated for dried fruits, but in general the order of magnitude is in the same range. Ognik and colleagues investigated Aronia berries harvested in two different years as well as from different places, i.e., an unpolluted and polluted site [21]. Whereby no influence of pollution was observed for Cd, Pb was found with four times higher concentration in the berries grown on polluted soil. Considering water content of 80%, the results for lead by the Polish working group and those from the presented study are in good agreement. Comparing Aronia berries with the fruits from *Crataegus monogyna* [22], also a plant of the Rosaceae family, it can be seen that the majority of elements are present in lower amounts. Cd, Fe, Mg, Na, and Pb are in the same concentration range. The content of Cu, Ni, and Sr is even higher in Aronia berries. Since soil composition, ripeness state, climate and environmental conditions and genetic background influence the accumulation of elements, these obtained differences are to be expected [24].

In Table 6 the Recommended Dietary Allowances (RDA) are given for selected essential elements [25]. A daily intake of 10 g dried berries would cover only 1.2%; 0.2%; 0.8%; 0.2%; 1.7%; 4%; 0.3% and 0.05% of the daily needs of Ca, Cr, Cu, Fe, Mg, Mn, Mo, and Zn, respectively. From these values the conclusion can be drawn that Aronia berries can only contribute to a small but appreciable amount of the dietary metal uptake. There is also no health risk posed by toxic elements in the analyzed samples, but for the general conclusion more samples from a different sampling location have to be measured. The Joint FAO/WHO Expert Committee on Food Additives (JECFA) proposes Provisional Tolerable Weekly Intake values (PTWI) for harmful elements in food: for Cd, Cr, Cu and Pb listed in Table 7 [26,27]. Considering a consumption of 100 g dried Aronia berries in per week and a body weight of 80 kg, the total intake in µg as well as in µg/kg body weight (BW) was calculated and the data are presented in Table 7. Only for Cu is the amount close to the given PTWI value (57%), whilst for the other toxic elements no negative health effect is to be expected (1.0%, 0.15% and 2.0% for Cd, Cr and Pb, resp.).

### 3.3. Elemental Analysis of Infusions

**Figure 1:**
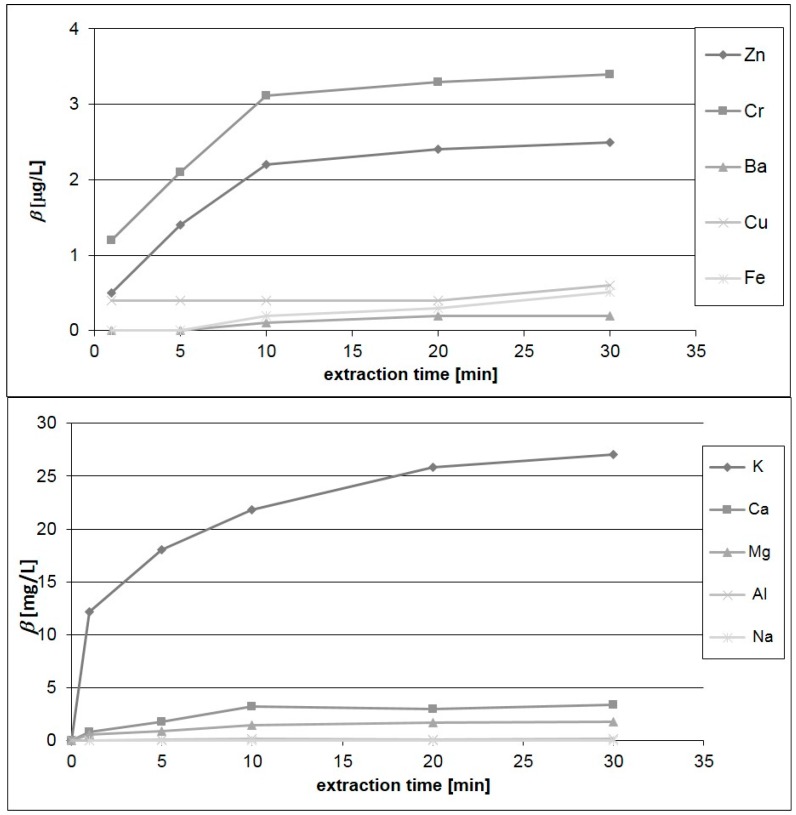
Concentrations of macro- and microelements in infusions after different extraction times.

Different decoctions times were applied in order to find the optimum one. The resulting metal concentrations from macro- and microelements depending on the extraction period ranging from 5–30 min are presented in Figure 1. For all elements more than 95% of the maximum extracted concentration is reached after 10 min, thus this period can be recommended for preparing tea from Aronia berries. Thus, the determined elemental concentrations for 10 min extraction time are used for further discussion and presented in Table 5 along with the standard deviation (*n* = 4). Since the remaining particulate matter in the infusions led to tube plugging of ICP-MS, not all analytes could be analyzed in the infusions, i.e., As, Li, Mo and Se are not addressed. Aqueous extraction of Aronia berries was also described by Pliszka and co-workers [23]. They mixed 200 mL of water at room temperature with 50 g of Aronia berries. These researchers focused only on selected essential elements. Their results for calcium iron, magnesium and zinc (Table 5) are much higher than those obtained in the present study. This was, on the one hand, due to a higher amount of plant matter per water and, on the other hand, due to other extraction conditions. Savikin and colleagues also studied infusions from Aronia berries, but only for the phenolic and anthocyanin content [6].

For all elements detected in the infusions the extraction yields were calculated according to the formula below and the values are listed in Table 5. They range from 4 to 44%. Thus, Aronia teas contain not only phenolic compounds of nutritional importance, but also essential elements.

$${\mathrm{extraction}\;\mathrm{yield}}_{x}\;\left[\%\right]=\frac{c_{x,\;\;infusion}\left[µg/L\right]\times \;V_{infusion}\;\left[L\right]}{m_{berry\;}\left[g\right]\times w_{x,\;\;berry\;}\left[µg/g\right]}$$

## 4. Conclusions

Aronia berries as well as infusions derived from them are a good dietary source of essential metals in addition to the organic compound contained.The nutrients Ca, K and Mg are present in the fruits (dried matter) at g/kg level, whereas the other elements are in the µg/kg to mg/kg range. As, Ba, and Se were below the limit of detection. Infusions of the chokeberries investigated are a good dietary source of essential metals especially regarding Ca, Cu, Fe, K, Mg, Mn, Na, Ni, and Zn. In addition to the known beneficial impact from its organic compounds, the mineral composition justifies its usage as nutritional supplement. The toxic elements present do not pose any health risk when berries or infusions are consumed.

## Figures and Tables

**Table 1:** ICP-AES operating conditions.

Instrument	Prodigy High Dispersive ICP
Spectrometer	High resolution Echelle polychromator Large format programmable array detector (L-PAD)
RF-Generator	40 MHz “free-running”
Output power	1.1 kW
Argon flow	Coolant:18 L/min, Auxiliary: 0.8 L/min, Nebulizer: 1 L/min
Peristaltic pump	1.0 mL min^−1^
Nebulizer	Pneumatic (glass concentric)
Spray chamber	Glass cyclonic
Plasma viewing	Axial
Replicates for each analysis run	3
Sample uptake delay	30 s

**Table 2:** Recoveries and certified data for analytes in CRM strawberry leaves (LGC7162).

Content [mg/kg]	Al *	As	Ba	Ca	Cd	Co	Cr	Cu *	Fe	K	Li *	Mg	Mn	Mo	Na *	Ni	Pb	Se *	Sr	Zn
found	1036	0.278	112	15,181	0.186	0.459	1.87	8.7	791	20,313	0.623	3829	162	0.300	218	2.30	1.78	0.347	63.8	21.5
certified value	1000	0.28	107	15,300	0.17	0.47	2.15	10	818	19,600	0.7	3770	171	0.32	210	2.6	1.8	0.04	64	24
confidence interval		0.07	10	700	0.04	0.11	0.34		48	1000		170	10	0.08		0.7	0.4		6	5
method	AES	MS	AES	AES	MS	MS	MS	MS	AES	AES	MS	AES	AES	MS	AES	MS	MS	MS	MS	AES
recovery in %	104	99	105	99	109	98	87	87	97	104	89	102	95	94	104	85	99	87	100	90

* only indicative value for this element.

**Table 3:** Recoveries for analytes in infusions.

Concentration [mg/L]	Al	Ba	Ca	Cd	Co	Cr	Cu	Fe	K	Mg	Mn	Na	Ni	Pb	Sr	Zn
spiked	1	1	5	1	1	1	1	3	5	3	1	5	1	1	1	1
found	1.02	1.03	4.87	1.06	1.04	1.02	0.976	2.84	5.12	3.08	0.957	4.73	0.981	1.02	0.924	0.976
recovery in %	102	103	97	106	104	102	98	95	102	103	96	95	98	102	92	98

**Table 4:** Elemental composition of Aronia berries in mg/kg dried matter (mean values and standard deviation, SD; *n* = 11).

Sample/Method	Determined Value	Al	As	Ba	Ca	Cd	Co	Cr	Cu	Fe	K	Li	Mg	Mn	Mo	Na	Ni	Pb	Se	Sr	Zn
Aronia berries	LOD [mg/kg]	0.91	0.020	0.86	0.12	0.0005	0.0037	0.0068	0.0072	0.05	0.27	0.0085	0.21	0.32	0.011	0.059	0.0063	0.013	0.0047	0.06	0.35
analytical method	-	AES	MS	AES	AES	MS	MS	MS	MS	AES	AES	MS	AES	AES	MS	AES	MS	MS	MS	MS	AES
Aronia berries	content [mg/kg]	158	^a^	^a^	1212	0.055	0.019	0.029	1.58	1.32	6790	0.012	669	0.829	0.039	4.27	0.38	0.041	^a^	1.66	0.55
	SD [mg/kg]	72			310	0.027	0.001	0.018	0.07	0.05	308	0.001	89	0.123	0.016	1.22	0.02	0.021		0.40	0.13
Aronia berries [11]	content in fresh fruits [mg/kg]				272						2903		155								
Aronia berries [20] reported in [3]	content in fresh fruits [mg/kg]				322					9.3	2180		162			26					1.47
Aronia berries [21]	content [mg/kg] unpolluted area (2003/04)					0.047/0.045												0.012/0.014			
(fresh fruits)	content [mg/kg] polluted area (2003/04)					0.047/0.046												0.043/0.048			
Hawthorn berries [22]	content [mg/kg]	917	0.012	4.17	3722	0.045	0.058	1.31	0.55	2.4	4223	0.51	987	1.88	0.097	6.34	0.067	0.027		0.41	1.12

^a^ <LOD (limit of detection).

**Table 5:** Elemental composition of infusions made from Aronia berries after 10 min decoction time (mean values and standard deviation, SD; *n* = 4; infusions based on 2 g dried berries + 98 mL water).

Sample/Method	Determined Value	Al	Ba	Ca	Cd	Co	Cr	Cu	Fe	K	Mg	Mn	Na	Ni	Pb	Sr	Zn
infusions from Aronia berries	LOD [µg/L]	0.58	0.09	2.1	0.41	0.57	0.77	0.40	0.21	0.05	2.8	0.08	0.09	1.0	5.1	0.07	0.81
analytical method	^a^	AES	AES	AES	AES	AES	AES	AES	AES	AES	AES	AES	AES	AES	AES	AES	AES
infusions from Aronia berries	Concentration [µg/L]	160	0.10	3235	^b^	^b^	3.2	3.3	0.31	21,821	1485	0.40	8.7	2.3	^b^	^b^	2.2
	SD [µg/L]	14	0.04	89			0.4	0.03	0.02	264	72	0.02	0.3	0.1			0.1
	extraction yield in %	4.3	n.c.	7.6			25	27	9.5	11	7.2	4.0	44	20			23
extracts from Aronia berries 50 g berries in 200 mL solution [23]	concentration [µg/L]			29,530					620		37,490						2750

^a^ due to high organic and particulate matter (even after centrifugation) measurement of the infusions with ICP-MS was not possible; ^b^ < LOD (limit of detection) n.c. not calculated.

**Table 6:** Recommended Dietary Allowances (RDA) for adults for determined essential elements [25].

Element	RDA [mg/day]	Element	RDA [mg/day]
Ca	1000	Mg	400
Cr	0.120	Mn	2
Cu	2		
Fe	8	Mo	0.120
		Zn	11

**Table 7:** PTWI for determined harmful elements along with calculated intake by consumption of 100 g dried berries.

Element	PTWI [26,27] [µg/kg BW]	Mass in 100 g Dried Berries [µg]	Weekly Intake by Consumption of 100 g Dried Berries
Cd	7.0	5.5	0.069
Cr	23.3	2.9	0.036
Cu	3.5	158	2.0
Pb	25	4.1	0.051
